# Evidence of subtle genetic structure in the sympatric species *Mullus barbatus* and *Mullus surmuletus* (Linnaeus, 1758) in the Mediterranean Sea

**DOI:** 10.1038/s41598-017-18503-7

**Published:** 2018-01-12

**Authors:** Sanja Matić-Skoko, Tanja Šegvić-Bubić, Ivana Mandić, David Izquierdo-Gomez, Enrico Arneri, Pierluigi Carbonara, Fabio Grati, Zdravko Ikica, Jerina Kolitari, Nicoletta Milone, Paolo Sartor, Giuseppe Scarcella, Adnan Tokaç, Evangelos Tzanatos

**Affiliations:** 1 Institute of Oceanography and Fisheries, Šetalište I. Meštrovića 63, 21000 Split, Croatia; 2 Production Biology Department, Nofima AS, P.O. Box 6122, 9291 Tromsø, Norway; 3 FAO AdriaMed Project, Viale delle Terme di Caracalla, 00153 Roma, Italy; 4 COISPA Tecnologia & Ricerca - Stazione Sperimentale per lo Studio delle Risorse del Mare, Via dei Trulli 18/20, 70126 Bari, Italy; 5 ISMAR-CNR, Institute of Marine Sciences of the Italian National Research Council, Largo Fiera della Pesca 2, 60125 Ancona, Italy; 6 Institute of Marine Biology, University of Montenegro, P. Fah 69, 85330 Kotor, Montenegro; 7 Aquaculture and Fishery Laboratory, Agricultural University of Tirana, Tirana, Albania; 8 CIBM Centro Interuniversitario Biologia Marina ed Ecologia Applicata “G. Bacci”, Viale Nazario Sauro 4, 57128 Livorno, Italy; 9 Ege University, Faculty of Fisheries, 35100 Bornova, Izmir, Turkey; 10 Department of Biology, University of Patras, 26504, Patras, Greece

## Abstract

Using thirteen microsatellite loci for *Mullus barbatus* and *Mullus surmuletus* collected in the Mediterranean Sea, the biogeographic boundaries, genetic distribution among and within basins and the impact of prolonged exploitation in both species were investigated as a basis for understanding their population dynamics and for improving *Mullus* spp. stock management. Different level of diversity indices among these co-occurring species were obtained, with *M. barbatus* showing higher allele richness and higher mean observed and expected heterozygosity than *M. surmuletus*. Reduced contemporary effective population size (Ne) and M-ratio values found in both species likely reflects recent demographic changes, due to a combination of high fishing pressures, habitat fragmentation and naturally occurring fluctuations in population size. Different patterns of genetic connectivity among populations sampled within the Mediterranean were observed for both species. Higher genetic structure was found for *M. barbatus* as opposed to a more homogenous pattern observed in *M. surmuletus* samples. Adriatic populations, previously considered panmictic and isolated from other Mediterranean regions, showed geographical partitioning within the basin but also population connectivity with the northern Ionian and Tyrrhenian Seas. Our results highlight the need for temporal sampling in understanding the complex pattern of population connectivity in the Mediterranean, particularly for management purposes.

## Introduction

The striped red mullet (*Mullus surmuletus* L., 1758) and the red mullet (*Mullus barbatus* L., 1758) are distributed in the eastern Atlantic Ocean, from the North Sea to Senegal, and throughout the Mediterranean and Black Seas. Both species are demersal and share very similar biological characteristics, with the main distribution to depths of 200 m in the continental shelf. Goatfishes show bathymetric habitat partitioning and clear niche segregation in relation to the bottom type that constitutes their habitat^1^. However, recent studies have shown that depth alone is not as significant as previously thought, masking the influence of salinity and temperature. *M. barbatus* shows an affinity for low salinity waters and *M. surmuletus* for warmer waters, which may contribute to their spatial segregation^2^.

Both species are considered important resources for coastal Mediterranean demersal fisheries^3^, as they are among the most valuable species in commercial landings being fished simultaneously or sequentially using a number of gears that vary over the year. They are caught mainly by the trawling fleet^4^ and thus are subject to intense fishing pressure. The trawl fleets generate 80% of *Mullus* landings, with *M. barbatus* representing ≈70% of this fraction. Red mullet stocks are composed mainly of young fish^4^ and thus are highly susceptible to overfishing. However, in small–scale fisheries that account for the remaining 20% of total landings, *M. surmuletus* and *M. barbatus* are caught in the ratio 75:25%. It is known that goatfishes respond to human-induced factors such as fisheries and habitat modification, as reflected by abundance, size, or weight changes, or changes in their distribution ranges^5^. Furthermore, temperature increase may lead to increased reproductive or growth rates, and longer warming periods may induce goatfishes to migrate to higher latitudes^6^.

Stock definition and identification and spatial structure information provide a basis for understanding fish population dynamics, and play a key role in fisheries assessment and management^7,8^. Each stock has temporal or spatial integrity^9^ and may have unique demographic properties (growth, recruitment, mortality and fishing mortality) and responses or rebuilding strategies to exploitation. For fish stocks, as subpopulations of a particular species, intrinsic parameters are the only significant factors in determining stock dynamics, while other factors, in particular immigration and emigration, are considered to have limited effect. Biological attributes and productivity of species may be affected if the stock structure and fisheries management are not well considered^10^. Thus, stock identification is of primary importance for population structure assessment of economically important species.

In practice, it is not easy to identify stocks, as the delimitation of adjacent populations involves many issues, especially in the sea where there are no clear geographical barriers^8^. There are a variety of techniques for stock identification, including genetics, morphometry, biological descriptor (i.e. reproduction, growth), parasites and others^11^. However, most techniques give inconclusive results or are costly^12^. Therefore, fishery scientists delineate spatial entities, such as management units or geographic areas, for monitoring harvested stocks^8^ with biological, geographic, economic, social or even political factors used to outline these entities^7^. This is the case of the Mediterranean, where the General Fisheries Commission for the Mediterranean (GFCM) has established thirty management areas (Geographical subareas - GSA) based on political and statistical considerations, rather than on biological or economic factors^13^. Thus, the waters of Adriatic Sea were recognized as two independent sub-areas (GSA17 and GSA18). In some cases, stock assessments are performed joining two adjacent GSAs (e.g., for *M. barbatus* for GSAs 17 + 18 in 2014 or for GSAs 13 + 14 in 2015) based on previous stock assessments carried out at the national level, due to similar biological characteristics of the two populations of red mullet and similar changes in CPUE levels in those two GSAs^14^. However, a possible mismatch between the stock limits established by fishery managers and the biological stock boundaries can be expected. Moreover, it has been shown that stocks need not to be completely isolated to show demographic independence^15^.

Genetic studies have been carried out in the Mediterranean Sea on both species^16^, revealing highly structured metapopulations. A sharp genetic division was detected when comparing striped red mullet originating from the Atlantic Ocean and from Mediterranean Sea. In the Mediterranean basin, the Siculo-Tunisian Strait seems to mark a transition zone between the Mediterranean’s eastern and western populations^17^. However, a straightforward conclusion on heterogeneity among Mediterranean populations could not be provided, due to the relatively low number of populations sampled, limited sample sizes and limited number of nuclear markers examined, especially in case of the Adriatic Sea, where no complete genetic structure analysis of *Mullus* sp. was previously conducted. Namely, the isolation of the Adriatic populations of *M. barbatus* (as a panmictic unit) in the Mediterranean Sea has been recorded^18^ basing only on samples from the western Adriatic Sea and a sample off the Albanian coast. A lack of complete sampling design, where eastern Adriatic populations were not considered, may have resulted in a failure to identify discrete populations. Moreover, in the case of *M. surmuletus*, there is no concrete information on stock identity or spatial structure for the Adriatic Sea. Thus, the foundation for understanding their population dynamics is lacking, which disables reliable stock assessment for fisheries management.

In the present study, we investigated the genetic variability of two sympatric species *M. barbatus* and *M. surmuletus* in the Adriatic and Mediterranean Seas by using 13 microsatellite loci. The study aimed to: (i) clarify spatial genetic sub-structuring as a good base for improving *Mullus* spp. stock management; (ii) understand population genetic connectivity among and within basins affected by oceanographic features, and (iii) compare current effective population size (Ne) and occurrence of recent bottlenecks between the species in order to assess the impacts of constant fisheries pressure. Moreover, we argue that the defined GSAs within the Mediterranean cannot be maintained *a priori* as unique areas for assessment and management purposes for specific fish species, in this case, red mullets.

## Results

### Loci screening and within genetic diversity

**Figure 1:**
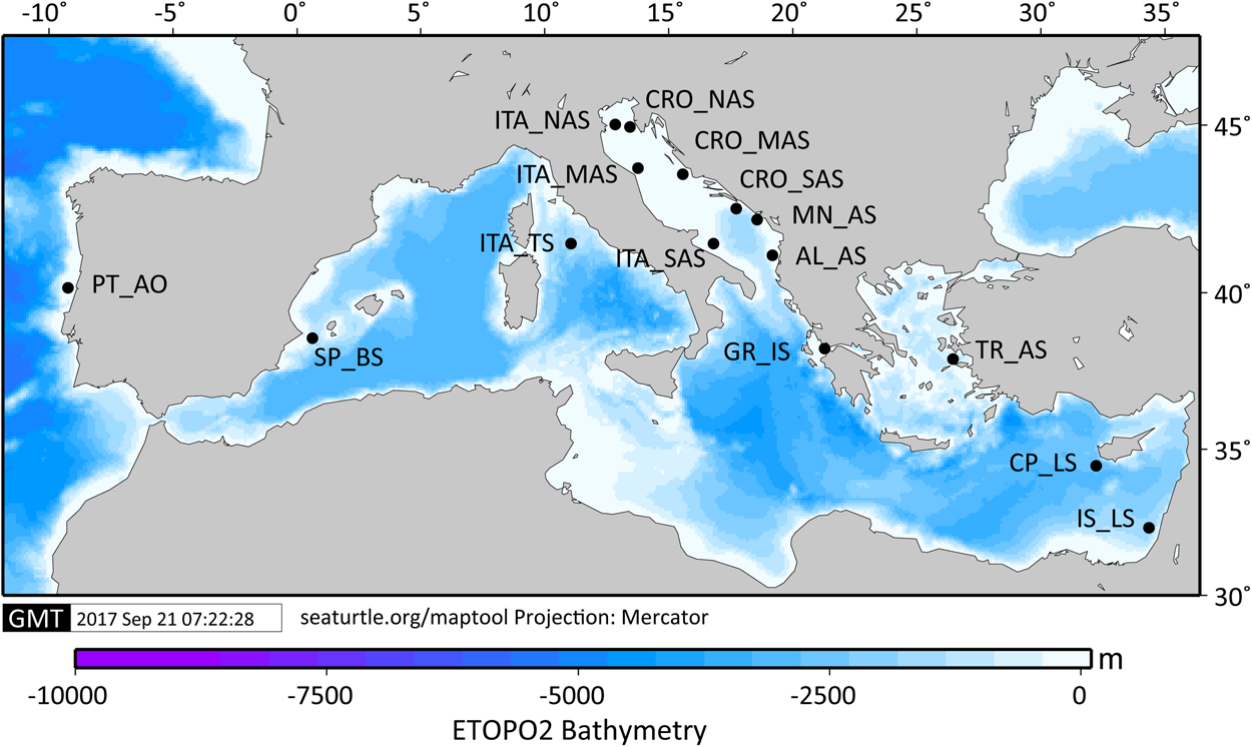
Geographic origin of the studied samples of *Mullus barbatus* and *Mullus surmulentus*. Sampling locations for both species: CRO_NAS, Croatia, north Adriatic; CRO_MAS, Croatia, middle Adriatic; CRO_SAS, Croatia, south Adriatic; ITA_MAS, Italy, middle Adriatic; ITA_SAS, Italy, south Adriatic; ITA_TS, Italy, Tyrrhenian Sea; MN_AS, Montenegro, south Adriatic; GR_IS, Greece, Ionian Sea; TR_AS, Turkey, Aegean Sea; IS_LS, Cyprus, Levantine Sea; SP_BS, Spain, Balearic Sea. Sampling locations for only *M. barbatus*: ITA_NAS, Italy, north Adriatic; AL_AS, Albania, south Adriatic. Sampling location for only *M*. *surmuletus*: PT_AO, Portugal, eastern Atlantic. More information about populations and regional subdivision are provided in the Table S1, Supplementary information. Map created using Maptool^82^.

A total of 720 individuals of *M. barbatus* and 599 individuals of *M. surmuletus* (sample sizes given in Tables 1 and 2, Fig. 1) were genotyped at 13 microsatellite loci. The proportion of missing data per locus ranged between 0 and 3.5%, with an average of 1.2% and 0.7% for *M. barbatus* and *M. surmuletus*, respectively.

**Table 1:** Summary statistics for genetic variation of red mullet (*Mullus barbatus*) in the Mediterranean Sea showing sample size (N), average number of alleles (A), effective number of alleles (Ae), allelic richness (Ar), expected (He) and observed (Ho) heterozygosity, fixation index (*F*_IS_) and effective population size (*N*_E_) for 11 microsatellite loci.

Pop code	N	Allele diversity			Heterozygosity		*F*_IS_	*N*_*E*_
		A	Ae	Ar	Ho	He		
CRO_NAS	48	14.2 ± 6.7	6.9 ± 5.7	10.5 ± 4.6	0.70 ± 0.9	0.75 ± 0.1	0.071	129 (35, 294)
CRO_MAS	66	14.3 ± 7.3	5.9 ± 4.1	9.8 ± 4.3	0.68 ± 0.1	0.74 ± 0.1	0.059	229 (77, ∞)
CRO_SAS	50	13.4 ± 7.4	5.5 ± 4.9	9.6 ± 4.3	0.70 ± 0.1	0.74 ± 0.1	0.054	193 (142, 295)
ITA_NAS	49	13.0 ± 5.9	6.2 ± 4.3	9.5 ± 4.1	0.73 ± 0.1	0.76 ± 0.1	0.035	344 (155, 504)
ITA_MAS	50	13.3 ± 6.6	6.3 ± 5.4	9.8 ± 4.4	0.67 ± 0.1	0.73 ± 0.1	0.069	391 (174, 1966)
ITA_SAS	47	13.4 ± 7.0	6.8 ± 5.8	10.1 ± 4.9	0.74 ± 0.1	0.77 ± 0.1	0.046	284 (140, 705)
ITA_TS	49	13.0 ± 7.4	6.4 ± 5.1	9.9 ± 4.8	0.68 ± 0.1	0.75 ± 0.1	0.061	396 (127, ∞)
MN_AS	87	15.2 ± 8.9	6.6 ± 5.8	9.8 ± 4.8	0.67 ± 0.1	0.75 ± 0.1	0.095*	384 (112, ∞)
AL_AS	67	14.4 ± 8.1	6.5 ± 5.6	9.7 ± 4.8	0.68 ± 0.1	0.74 ± 0.1	0.081	479 (189, ∞)
GR_IS	59	12.9 ± 6.4	6.1 ± 4.3	9.5 ± 3.8	0.69 ± 0.1	0.76 ± 0.1	0.066	425 (209, ∞)
TR_AS	48	13.6 ± 7.2	6.3 ± 4.3	10.1 ± 4.4	0.72 ± 0.1	0.76 ± 0.1	0.057	261 (26, ∞)
IS_LS	23	10.1 ± 4.1	5.7 ± 3.6	9.6 ± 3.8	0.67 ± 0.1	0.77 ± 0.1	0.131*	—
CP_LS	31	10.1 ± 3.9	5.5 ± 3.6	9.2 ± 3.8	0.66 ± 0.1	0.75 ± 0.1	0.124*	118 (47, 209)
SP_BS	46	13.3 ± 6.4	6.0 ± 4.2	9.8 ± 4.3	0.67 ± 0.1	0.74 ± 0.1	0.099*	159 (104, 311)
Overall	720	13.6 ± 6.7	6.7 ± 5.1	9.9 ± 4.2	0.70 ± 0.1	0.76 ± 0.1		

Effective population size (*N*_E_) of the population with small sample size (IS_LS) was not analysed.

**Table 2:** Summary statistics for genetic variation of striped red mullet (*Mullus surmuletus*) in the Mediterranean Sea showing sample size (N), average number of alleles (A), effective number of alleles (Ae), allelic richness (Ar), expected (He) and observed (Ho) heterozygosity, fixation index (*F*_IS_) and effective population size (*N*_E_) for 11 microsatellite loci.

Pop	N	Allele diversity			Heterozygosity		*F*_IS_	*N*_*E*_
		A	Ae	Ar	Ho	He		
CRO_NAS	50	11.8 ± 4.8	5.7 ± 3.5	7.2 ± 2.6	0.62 ± 0.2	0.73 ± 0.2	0.118*	138 (49, 245)
CRO_MAS	54	11.0 ± 5.5	5.4 ± 3.3	6.7 ± 2.7	0.67 ± 0.2	0.71 ± 0.2	0.056	106 (51, 752)
CRO_SAS	49	11.3 ± 5.7	5.6 ± 3.5	6.9 ± 2.8	0.70 ± 0.2	0.72 ± 0.2	0.029	100 (72,157)
ITA_MAS	47	10.2 ± 4.8	7.3 ± 2.9	6.7 ± 2.6	0.69 ± 0.2	0.72 ± 0.2	0.035	78 (51, 143)
ITA_SAS	42	10.2 ± 5.2	4.9 ± 2.8	6.5 ± 2.6	0.76 ± 0.2	0.72 ± 0.2	−0.057	113 (57, 686)
ITA_TS	45	11.0 ± 5.6	5.6 ± 3.9	6.8 ± 3.0	0.68 ± 0.2	0.72 ± 0.2	0.046	99 (70, 157)
MN_AS	31	11.0 ± 4.2	5.8 ± 3.6	7.3 ± 2.3	0.66 ± 0.2	0.76 ± 0.2	0.051	67 (26, 131)
GR_IS	59	12.2 ± 4.9	5.3 ± 2.9	6.8 ± 2.5	0.69 ± 0.2	0.72 ± 0.2	0.030	114 (69, 257)
TR_AS	49	10.5 ± 5.4	5.4 ± 3.5	6.6 ± 2.8	0.70 ± 0.2	0.72 ± 0.2	0.033	106 (64, 238)
IS_LS	15	6.2 ± 2.5	3.9 ± 1.9	5.5 ± 2.0	0.60 ± 0.2	0.65 ± 0.2	0.085	—
CP_LS	49	11.4 ± 4.8	5.4 ± 2.6	7.1 ± 2.6	0.65 ± 0.2	0.74 ± 0.2	0.102*	45 (16, ∞)
SP_BS	41	10.4 ± 5.3	5.3 ± 3.2	6.7 ± 2.8	0.66 ± 0.2	0.71 ± 0.2	0.081*	91 (49, 307)
PT_AO	68	11.7 ± 6.3	5.2 ± 3.1	6.6 ± 2.8	0.64 ± 0.2	0.68 ± 0.2	0.070*	157 (89, 452)
Overall	599	10.7 ± 5.0	5.9 ± 3.8	6.7 ± 2.6	0.67 ± 0.2	0.71 ± 0.2		

Effective population size (*N*_E_) of the population with small sample size (IS_LS) was not analysed.

The majority of *Mullus barbatus* populations showed significant deviation from Hardy-Weinberg equilibrium, with tendencies towards heterozygote deficiency at the loci Mbar3, Mbar130, Mbar101 and Mbar63 (Table S2a, Supplementary information), as revealed by Fisher’s exact test. MICROCHECKER identified these loci as potentially exhibiting null alleles with the estimated frequencies of null alleles <0.3 for Mbar130 and Mbar101. More specifically, the analysis indicated homozygote excess at loci Mbar130 and Mbar101 for most allele size classes. Those two loci were removed from further analyses. Much rarer null alleles at Mbar3 and Mbar63 were detected via MICROCHECKER but had an estimated global frequency of only 0.04 and 0.07, respectively. Genotype data were retained, since the estimation of F_ST_ both using and without using the ENA correction method gave equal results; F_ST_ = 0.027 with the respective 95% CI [0.003–0.028] without the ENA and [0.003–0.027] with the ENA. Population differentiation parameters are only slightly biased with a null allele frequency ranging between 5% and 8% on average across loci^19^. No consistent evidence for linkage disequilibrium was detected between pairs of loci within populations.

For *Mullus surmuletus*, significant deviation from Hardy-Weinberg equilibrium was observed in certain populations (CRO_NAS, CRO_MAS, MN_AS, CP_LS), with tendencies towards heterozygote deficiency at the loci Mbar130 and Mbar133 (Table S2b, Supplementary information). MICROCHECKER identified these loci as potentially exhibiting null alleles with the estimated frequencies of null alleles <0.2. Both loci were discarded from future analysis. Linkage disequilibrium was not detected between pairs of loci within populations.

Among the 11 loci examined, all were polymorphic, with the number of alleles per locus ranging from 2 to 27 for *Mullus barbatus* and 2 to 24 for *Mullus surmuletus*. Genetic diversity revealed varying degrees of genetic diversity among populations, ranging from 0.67 to 0.74 in expected heterozygosity (*H*_e_) in *M. barbatus* and from 0.60 to 0.76 in *H*_e_ in *M. surmuletus* (Tables 1 and 2). More specifically, striped red mullet exhibited significantly lower effective number of alleles per locus (5.9 *vs* 6.7), allelic richness (6.7 *vs* 9.9) and observed heterozygosity (0.67 *vs* 0.70) (ANOVA test, p < 0.05) in comparison to red mullet. No obvious geographical patterns were observed for the distribution of genetic diversity, except for the sampling site ITA_SAS (off the southern Italian coast), where the level of observed heterozygosity for both species was highest of all sites. The inbreeding coefficient, *F*_IS,_ ranged from 0.035 to 0.131 in the red mullet dataset, and striped from −0.057 to 0.118 in striped red mullet, respectively, and was significantly higher than zero in 4 of the total 14 and 13 populations of both fish species (Tables 1 and 2).

The estimated contemporaneous effective population size, *N*e, of red mullet ranged from 129 (CRO_NAS) to 479 (AL_AS) where the upper confidence limit reached infinity in some cases (6 of 13 populations). The *N*e of striped red mullet ranged from 67 (MN_AS) to 157 (PT_AO) with upper and lower 95% CI of 26 and 686 (Tables 1 and 2). On average, estimates of Ne were threefold smaller in striped red mullet (95) than in red mullet (292) populations.

**Figure 2:**
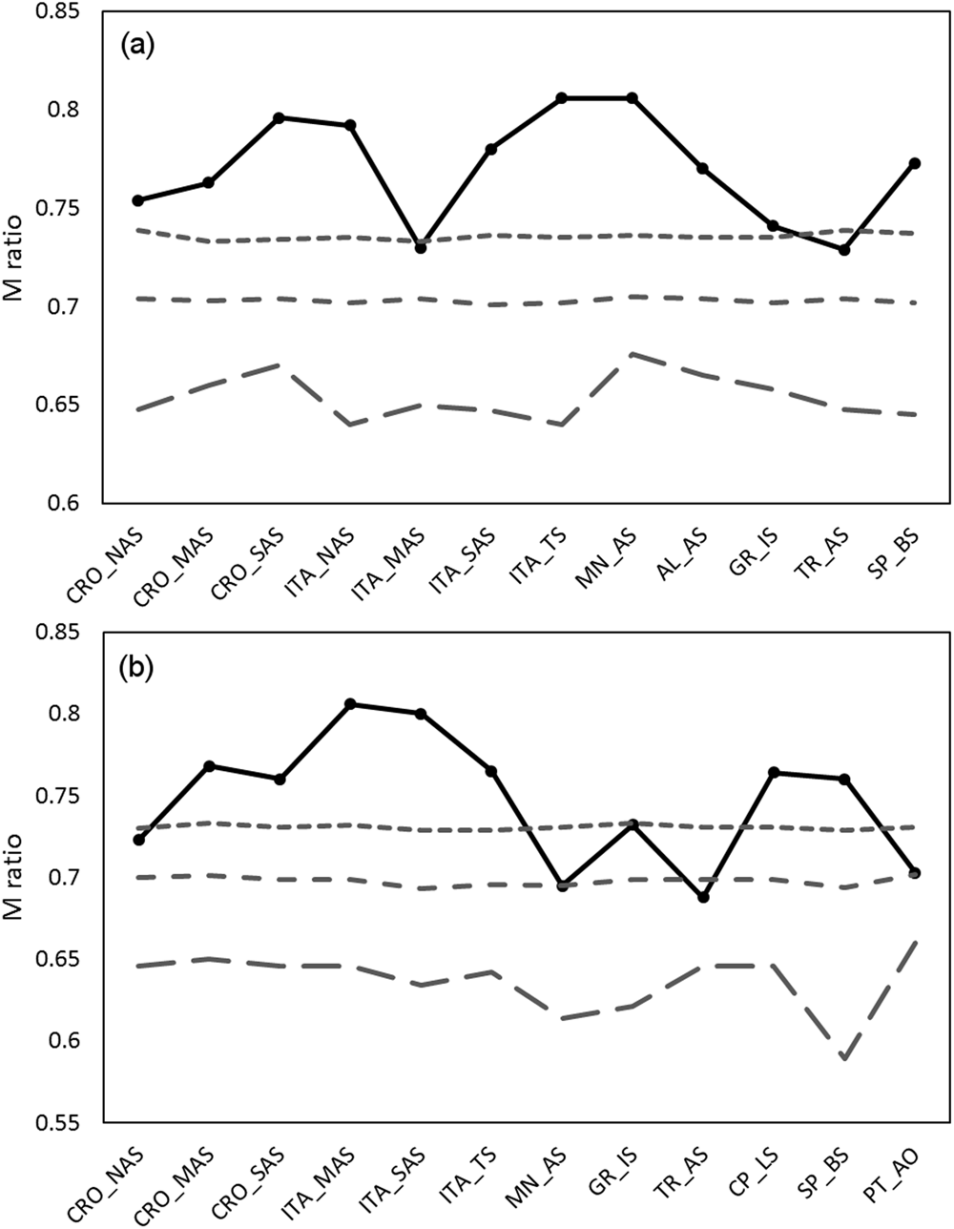
Observed and simulated values of the Garza-Williamson index for (**a**) *Mullus barbatus* and (**b**) *Mullus surmuletus* populations. The observed *M*-ratio values are displayed in black solid line. The simulated critical *M* threshold (*M*c) values below which a bottleneck is evident, was calculated for different ancestral theta and displayed by grey dot (θ = 0.5), dash (θ = 1) and long dash (θ = 10) lines. Populations *M*-ratio and the *M*c parameters were calculated using M-P-Val and Critical_M^39^.

Both bottleneck tests showed statistical evidence that red mullet populations from the Ionian Sea (Greece) and Aegean Sea (Turkey) had undergone a recent reduction in population size. Wilcoxon signed-rank tests detected significant heterozygote excess (p < 0.05) under the infinite alleles model for each of these populations, while the observed M ratios were significantly lower than the simulated equilibrium distribution of M for all pre-bottleneck values (P < 0.001, Table S3a, Supplementary information). Additionally, the population ITA_MAS from the middle west Adriatic showed evidence of a bottleneck by this method, but only for ancestral theta (θ) values of 0.5. Estimated M values for all other populations (0.754–0.806) were above their critical M values (Fig. 2a).

The observed M ratios for three populations of striped red mullet (CRO_NAS, MN_AS and PT_AO) were significantly lower than the simulated equilibrium distribution of M for the ancestral θ value of 0.5 (P < 0.03, Fig. 2b), while for the Aegean Sea population (TR_AS), the M value was significantly lower in cases of 0.5 and 1 tested θ (0.688, P < 0.04). TR_AS also demonstrated significant heterozygote excess under the infinite alleles model (P < 0.01), showing evidence of recent genetic bottleneck (Table S3b, Supplementary information).

### Among-Population Genetic Differentiation

Assessment of the statistical power for both microsatellite data sets in POWSIM revealed that it was possible to detect genetic divergence as low as *F*_ST_ = 0.01 with 100% certainty (χ^2^, Fisher’s test) and with 57% *(M. barbatus*) and 35% (*M. surmuletus*) certainty for *F*_ST_ = 0.001.

At the broad Mediterranean scale, the overall *F*_ST_ value was 0.027 (95% CI = 0.014 to 0.042) for red mullet and 0.011 (95% CI = 0.007 to 0.016) for striped red mullet, and both values were highly significant (p < 0.001), supporting the spatial heterogeneity. Pairwise *F*_ST_ across all samples of red mullet ranged from –0.030 to 0.084 (Table 3a), with 53 of 91 pairwise comparisons at p < 0.01 when permuted by Fisher’s exact test. The majority of non-significant comparisons were found within populations from the northern and middle Adriatic Sea, including populations from both the western and eastern sides. These populations paired with the samples from the Tyrrhenian Sea, where *F*_ST_ ranged from 0.001 to 0.005. On the contrary, in the southern Adriatic, populations from the eastern Adriatic coast (CRO_SAS, MN_AS, AL_AS) showed a break in gene flow toward the north and middle Adriatic regions and further to the south, including the Ionian Sea (0.024 < *F*_ST_ < 0.049). No significant pair-wise differentiations were observed for populations within the eastern Mediterranean, while reduced gene flow was noted between the western Mediterranean region (Balearic Sea) and Adriatic Sea, but not between the western and eastern Mediterranean.

**Table 3:** Pairwise *F*_*ST*_ values among populations of (a) red mullet (*Mullus barbatus*) and (b) striped red mullet (*Mullus surmuletus*) from the Mediterranean Sea.

(a)	CRO NAS	CRO MAS	CRO SAS	ITA NAS	ITA MAS	ITA SAS	IT TS	MN AS	AL AS	GR IS	TR AS	IS LS	CP LS
CRO_MAS	0.005												
CRO_SAS	0.035	0.049											
ITA_NAS	0.004	0.003	0.040										
ITA_MAS	0.004	0.004	0.036	0.006									
ITA_SAS	0.008	0.003	0.030	0.003	0.003								
ITA_TS	0.002	0.003	0.054	0.004	0.001	0.004							
MN_AS	0.022	0.029	0.077	0.026	0.023	0.029	0.024						
AL_AS	0.021	0.031	0.081	0.026	0.022	0.030	0.024	−0.001					
GR_IS	0.001	0.002	0.061	0.002	0.004	0.001	0.003	0.022	0.024				
TR_AS	0.019	0.027	0.069	0.022	0.023	0.026	0.020	−0.001	−0.002	0.021			
IS_LS	0.016	0.027	0.069	0.022	0.022	0.026	0.021	−0.003	0.000	0.019	−0.004		
CP_LS	0.005	0.014	0.050	0.007	0.007	0.015	0.010	−0.023	−0.022	0.001	−0.029	−0.017	
SP_BS	0.027	0.038	0.084	0.031	0.032	0.037	0.032	−0.001	0.001	0.029	0.001	−0.003	−0.023
**(b)**	**CRO NAS**	**CRO MAS**	**CRO SAS**	**ITA MAS**	**ITA SAS**	**IT TS**	**MN AS**	**GR IS**	**TR AS**	**IS LS**	**CP LS**	**SP BS**	
CRO_MAS	0.004												
CRO_SAS	0.012	0.009											
ITA_MAS	0.004	0.003	0.009										
ITA_SAS	0.001	0.002	0.010	0.002									
ITA_TS	−0.001	0.002	0.011	0.002	−0.001								
MN_AS	0.013	0.021	0.011	0.022	0.016	0.012							
GR_IS	−0.001	−0.001	0.008	0.001	−0.000	0.002	0.006						
TR_AS	0.003	0.003	0.013	0.007	0.008	0.005	0.007	0.004					
IS_LS	0.053	0.037	0.052	0.044	0.039	0.046	0.064	0.044	0.049				
CP_LS	0.009	0.004	0.016	0.008	0.004	0.006	0.018	0.006	0.008	0.032			
SP_BS	0.006	0.001	0.012	0.003	0.000	0.001	0.015	0.002	0.004	0.035	0.004		
AT_AO	0.035	0.029	0.027	0.029	0.017	0.019	0.037	0.018	0.036	0.037	0.010	0.011	

Significant *F*_*ST*_ values are underlined (at p < 0.01). Population codes explained in Tables S1 and S2 Supplementary information.

Overall, genetic differentiation of the striped red mullet was half that of red mullet. Pairwise *F*_ST_ ranged from −0.001 to 0.064 (Table 3b), with 43 of 78 pairwise comparisons at p < 0.01. The significant values were related with Atlantic-Mediterranean population comparisons and, as in red mullet, between south-eastern Adriatic and neighbouring populations, including those from the eastern Mediterranean. The significant *F*_ST_ values related with the Israel population should be taken with consideration due to the limited sample size.

**Figure 3:**
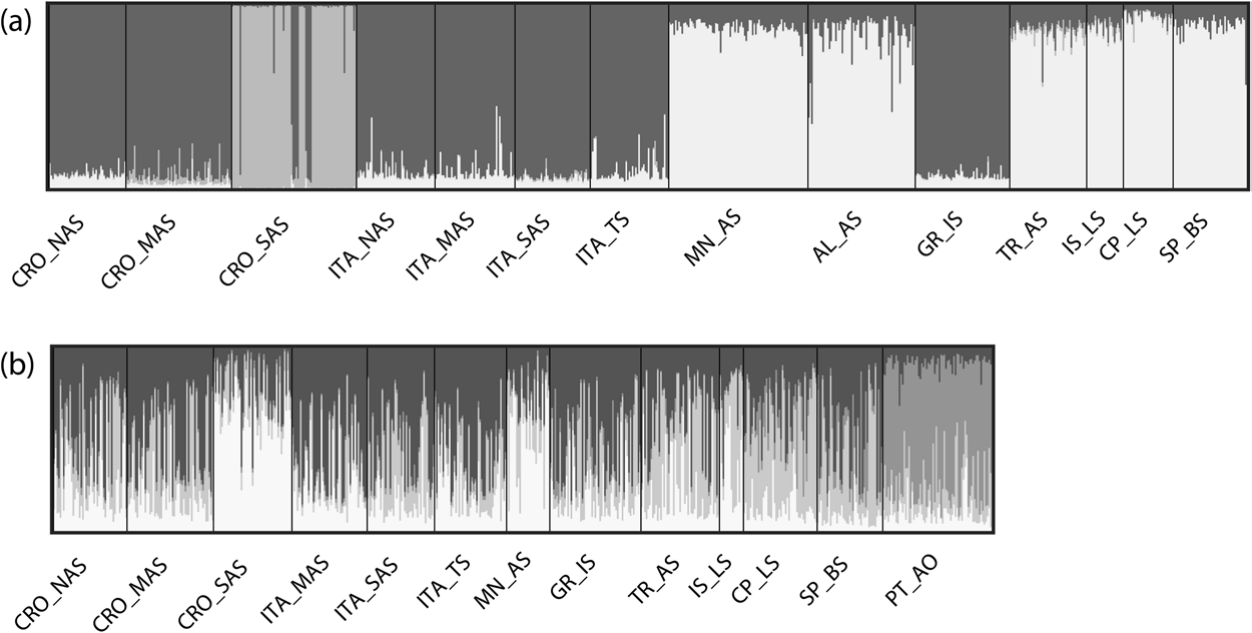
Bayesian clustering of (**a**) *Mullus barbatus* and (**b**) *Mullus surmuletus* populations according to STRUCTURE assignment scores, assuming three (K = 3) and four (K = 5) inferred clusters. See Tables 1 and 2 for abbreviated sample names.

**Figure 4:**
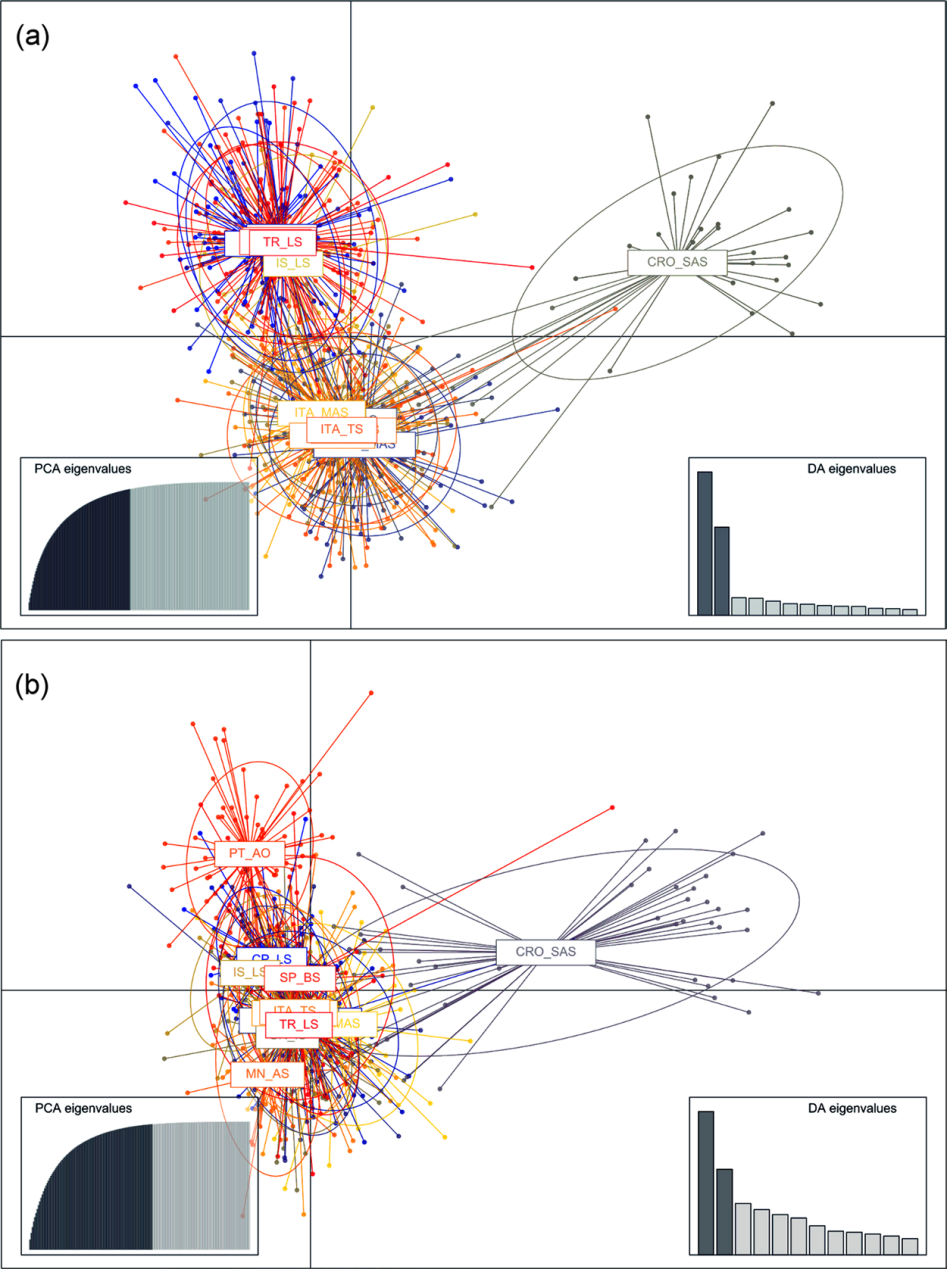
Discriminant Analysis of Principal Components (DAPC) of (**a**) *Mullus barbatus* and (**b**) *Mullus surmuletus* microsatellite genotypes using population/sample site as a group prior. The sample origin is labelled within their 95% inertia ellipses and individuals are connected to the corresponding group centroids.

The Bayesian clustering analysis of red mullet revealed three discrete genetic clusters that were supported by the mean likelihood score (Ln(K)) and the Delta K method (Fig. 3a, Fig. S1 Supplementary information). All three clusters were observed within the Adriatic Sea, separating the north and middle Adriatic populations (first cluster) from the south-eastern CRO_SAS population (second cluster) and the Montenegro-Albania populations (third cluster), with a high individual assignment score assigned to the specific cluster (Fig. S2 Supplementary information). In accordance with *F*_ST_ pair-wise results, no gene barrier was observed between the Balearic Sea and eastern Mediterranean, grouping all populations into the third cluster. Interestingly, the population from the Tyrrhenian Sea was assigned into the first cluster, together with the north and middle Adriatic populations. The DAPC plot clustered groups following the defined structure observed in STRUCTURE (Fig. 4a).

Examination of likelihood scores produced by STRUCTURE for the striped red mullet showed that the mean likelihood score (Ln(K)) plateaued at K = 4, while the Delta K method indicated K = 6 (Fig. S1 Supplementary information). However, increasing the number of clusters beyond *K* = 4 was not informative in revealing any additional population clusters, and instead only forced certain populations to accommodate even more admixture. The admixture bar plot for K = 4 demonstrated a less divergent spatial pattern among sampling sites in comparison to that observed in red mullet populations (Fig. 3b). Heterogeneity in cluster stratification showed that there was a trend for only two samples from the south-eastern Adriatic (CRO_SAS and MN_AS, Fig. S2 Supplementary information) and one from Atlantic (PT_AO) to be differentiated from the remaining sites. Namely, all other samples from the Mediterranean appeared to be a mixture of the three main clusters, while the forth cluster exclusively featured the Atlantic population. Mean cluster proportions across iterations showed that CRO_SAS and MN_AS were 65% and 52% assigned to Cluster 1 (white; Fig. 3b), while samples from the Atlantic Sea were >65% assigned to Cluster 2 (grey; Fig. 3b). The results of DAPC analysis deviated slightly from the STRUCTURE analyses, such that the first principal component emphasized genetic difference among CRO_SAS and all other populations, while the second principal component reflected differences between the Atlantic-Mediterranean regions, with partial sample overlap (Fig. 4b).

**Figure 5:**
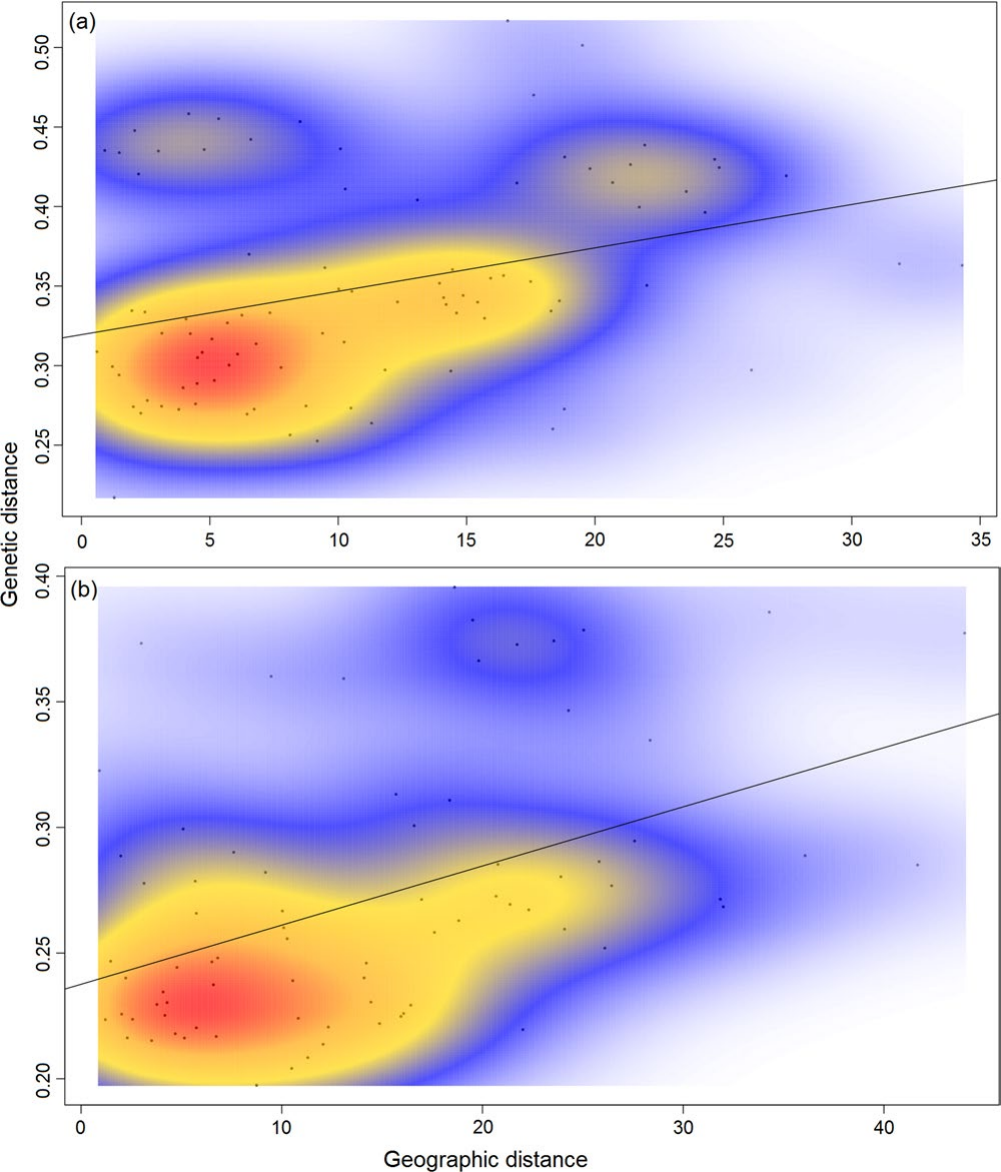
Isolation by distance plot illustrating the pattern of genetic differentiation in (**a**) *Mullus barbatus* (r = 0.33, *p* = 0.06) and (**b**) *Mullus surmuletus* populations (r = 0.453, *p* = 0.041) in respect to the pairwise geographical distances, using a two-dimensional kernel density estimation in MASS package in R.

The isolation by distance (IBD) analysis for the striped red mullet populations revealed a moderate isolation by distance pattern (r = 0.453, *p* = 0.041), where the scatterplot of local densities of distances showed only one consistent cloud of points, indicating a continuous cline of genetic differentiation (Fig. 5b). A less pronounced and marginally non-significant isolation by distance pattern (r = 0.33, *p* = 0.06) was detected for the red mullet populations. However, the scatterplot of local densities of distances indicated a patched pattern of genetic differentiation among populations, due to the observed discontinuity of one single consistent cloud (Fig. 5a).

## Discussion

Current practices aim to integrate genetic data of population structure into fisheries management strategies, considering that the sustainability of spawning stock biomass and the conservation of genetic diversity are predominantly linked to the evolutionary criteria of populations^7^. The basic unit for conservation, management, and sustainable use is a genetically homogenous group of individuals. Still, the degree of connectivity among marine taxa separated by biogeographic barriers can vary from extensive isolation to complete panmixia, and the differences observed are largely dependent on early life history traits, such as pelagic larval dispersal, as the presumed mechanism of primary connectivity^17,20^, but also the growth and reproduction characteristics. In this study, we evaluated large and fine-scale population processes of red mullet and striped red mullet from large distributional species ranges to seek a deeper understanding of the factors shaping genetic population structure at the broad Mediterranean scale and more locally, within the Adriatic Sea.

Three main findings can be observed from the present study. First, we found different levels of diversity indices among these co-occurring species, where *M. barbatus* showed a higher allele richness and higher mean observed and expected heterozygosity in contrast to *M. surmuletus*. This corroborated previous reports^17,21,22^, even though those studies employed only seven of the eleven microsatellite markers used in this study. Second, reduced contemporary Ne and M-ratio values found in both species likely reflects recent demographic changes due to a combination of high fishing pressures^23^, habitat fragmentation^24^ and naturally occurring fluctuations in population size^4^. Third, different patterns of genetic connectivity among populations sampled within the Mediterranean was observed for both species. Higher genetic structure was found for *M. barbatus* in contrast to the more homogenous pattern observed in *M. surmuletus* samples. Adriatic populations, previously considered to be panmictic and isolated from the rest of Mediterranean regions^18,25^, showed geographical partitioning within the basin but also population connectivity with the northern Ionian and Tyrrhenian Seas.

All recent molecular studies of *M. surmuletus* have been conducted using cross-specific microsatellites developed and characterized on *M. barbatus*, without previous empirical studies to highlight the impact of their use when quantifying levels of genetic variability. Namely, cross-specific amplification using highly polymorphic markers produces a biased picture of genetic diversity when compared with randomly specific markers^26^, which is often seen as an artificial decrease in polymorphism that occurs during transferability to non-focal species phase, with mutations either in the flanking region or in the repeat sequence^26,27^. In this data set, two loci (Mbar64 and Mbar3) developed for *M. barbatus* appear to exhibit evidence of a transfer bias, in which *M. surmuletus* had 50% reduced levels of *H*e than those described in the focal species (Table S2, Supplementary information). Thus, the lower genetic diversity and effective population size observed in *M. surmuletus* populations in comparison to the focal species could be a consequence of cross-specific amplification, that might lead to an ascertainment bias in multilocus heterozygosity estimates, as noted previously in genetic studies on *Merluccius paradoxus* and *M. capensis*^28^ and other animals^26,27^. However, it has been demonstrated that specificity and strategy of marker selection have no effect on population differentiation and clustering analysis in populations with lower genetic diversity^26^, highlighting the fact that assignment of individuals to populations could be affected if only a small number of randomly selected microsatellites are applied.

On the other hand, species that are heavily exploited and have overlapping generations, such as *M. surmuletus*, are predicted to show a faster decrease in genetic diversity^23^. Recent stock assessment points out that stocks of *M. surmuletus* are considered overfished, and in some Mediterranean sub-regions over-exploited (GSA05–GSA07, GSA09, GSA25)^29^. Strong fluctuations in population size due to fisheries negatively affect the effective population size, thus increasing the effects of selection and genetic drift, which could lead to lower genetic diversity levels, as observed in this study.

Both *Mullus* species showed reduced contemporary Ne in the order of several hundred (with or without Mbar64 and Mbar3 loci in *M. surmuletus* data set, data not shown). Such a pattern has previously been observed in several exploited marine fish species^10,23,30–32^. In addition to fishing pressures and habitat fragmentation, a strong bias in reproductive success, naturally variable recruitment patterns and size-dependent fecundity may additionally reduce the effective population size, in the sense that millions of individuals may be equivalent to an effective population size of only hundreds or thousands^30,33^. However, these interpreting Ne estimates for species with overlapping generations should be viewed cautiously, due to Wahlund effects caused by multiple cohorts in a sample^34^. Also, the heterozygosity deficit and significant departures from HWE observed in both *Mullus* species at some loci but not systematically for each location could be explained by population sub-structuring or selection, as previously reported by several authors^17,18,21,22^. Unnatural selection due to fisheries may induce evolution toward slow growth, early maturation at small size and higher reproductive investment^35^. Increased trend in the mean size related to lower fishing exploitation was observed for red mullet in the Castellamare Gulf, Sicily^36^, however long-term data series for other Mediterranean stocks lack further support for the above findings.

Neither bottleneck test used in the study identified coherent signals of genetic erosion, though there were some indications of demographic changes in certain populations. Significant heterozygote excess in the populations from Ionian and Aegean Seas was detected under the IAM mutation model, potentially corresponding to a bottleneck signal. However, the extent to which the IAM model truly describes the process of microsatellite mutation is questionable^37^. Still, evidence from M ratio tests, supposed to be more powerful at detecting bottlenecks compared to the heterozygosity excess test^38^, suggested that both *Mullus* species from the Aegean Sea may have experienced population bottlenecks. Furthermore, for two populations of *M. surmuletus* (MN_AS, TR_AS), M ratios were below the diagnostic value for genetic bottlenecks (0.70)^39^, while all sampled populations from both *Mullus* species had an M ratio less than 0.82, as the cutoff value for selecting demographically stable natural populations^39^. Even though interpretations of *M* values are not consistently supported across all the values of θ used^40^, these results might carry alarming signals that should not be overlooked.

Temporal or spatial fishing restrictions in some areas of the Mediterranean have demonstrated positive effects on *Mullus* sp. stock recovery through the increase of the size of the spawning fraction of population with a consequent increase on the recruitment success and spawner biomass^36,41,42^ or by promoting the admixture of individuals and increase of heterozygosity levels^21^. In the present study, we noted the greatest observed heterozygosity levels and the lowest values of inbreeding coefficients in both *Mullus* populations from the Italian side of the southern Adriatic Sea, which regionally coincides with nursery areas identified by means of indicator kriging^43,44^. Thus, nearby marine protection areas, such as Isole Tremiti and Torre Guaceto, play an important role in the protection of recruitments and preservation of the gene pool and genetic diversity of species. It is very interesting to note that according to last *M. barbatus* GFCM assessments^45^, GSA 18 diagnosis of stock status was sustainable exploited, supporting further our findings of greater observed heterozygosity level and the lower value of inbreeding coefficient within the region (Table S4, Supplementary information).

Different patterns of population structure were seen between the investigated species. While *M. barbatus* showed a greater level of structuring (*F*_ST_ = 0.027) when compared to its congener (*F*_ST_ = 0.011), both species showed significant population divergence within the Adriatic Sea localized in area of the South Adriatic Pit – the deepest part of the basin. In line with previous reports^17,18^, inconsistent genetic structure related with the major oceanographic barriers was observed for both species. While the main source of genetic differentiation of striped red mullet was the Atlantic-Mediterranean divide and Otranto Strait, red mullet populations were characterized with a puzzle pattern of gene flow discontinuity among the Sicily Channel, Otranto Strait and Aegean Front, with exclusion of the isolation-by-distance structure. Namely, red mullet from the Balearic Sea was significantly different from north and middle Adriatic populations, including the population from the Ionian Sea, whereas the opposite was noted for interactions among populations from the south Adriatic and eastern Mediterranean. Low values of *F*_ST_ and high gene flow in marine species does not necessary imply an absence of population structure^46^. Considering that few individuals per generation can maintain genetic homogeneity among populations^47^, more sampling is needed along the Mediterranean African coast, as well in the Sicily Channel and around Sardinia, to obtain representative patterns of genetic structure across a wide distribution range of the species. By using different markers, some authors confirmed genetic structuring at a relative small scale (RAPDs^48^) and also at a large scale, showing a structured population across the western and eastern Mediterranean Sea (allozymes^49^). Overall, the present study supports the findings of highly structured genetic distribution for red mullet^17^, where metapopulations are composed of independent, self-recruiting subpopulations with some connections between them.

A Mediterranean-Atlantic division of striped red mullet detected by microsatellite-based pairwise F_ST_ values has also been reported^17^ in a study that covered a large portion of the Atlantic-Mediterranean species distribution. The Almeria-Oran Front, the best-studied oceanographic discontinuity in the Mediterranean Sea, restricts gene flow in many other demersal and pelagic fish species^22,50,51^. Still, Bayesian analyses assigned the population from Portugal (Atlantic origin) to a separate cluster only partially (q > 65%), suggesting that adult dispersal and passive transport of eggs and larva occasionally occurs along the Strait of Gibraltar, likely due to interannual variability of the front that allows some degree of homogenization between localities^52^. As corroborated by the significant isolation by distance and continuous cline of genetic differentiation, the lack of strong genetic structure is largely the result of long distance larval dispersal (more than 400 km) by offshore currents, and the early life traits that differ slightly from those observed in red mullet^21^. Apart from spatial segregation of two sympatric species in relation to habitat use, where striped red mullet prefers shallower depths (10 to 50 m) and rough bottoms, while red mullet is most abundant on muddy bottoms in waters between 50 and 200 m deep^1^, the spawning and the recruitment of both species are temporally separated. Striped red mullet reproduction begins in March with a peak in April to May, and recruitment toward the bottom from August onwards, while red mullet reproduces from April to July with a peak in June and recruits toward the bottom in September and October^3,36,42,53,54^. Relatively small changes in water temperature have the potential to greatly influence patterns of fish growth and pelagic larval duration (PLD). It has been demonstrated experimentally that a 5 °C increase in water temperature decreased the time to metamorphosis by 2.8 d for the tropical species *Upeneus tragula* reared from midway through their pelagic life phase^55^. Similar observations were recorded for temperate flatfishes^56^. Thus, it could be argued that both *Mullus* species have a similar pelagic larval duration (28–35 days)^57^, with records based on very limited sample size (30–50 individuals), due to temperature differences between spring and summer seasons. In the case of striped red mullet, lower temperatures can prolong planktonic duration and dispersal ability, influencing genetic homogenization at a wider geographical scale^6^. Still, the impact of batch spawning and different spawning pulses during the reproductive season^42^, in contrast to the larval prolonged competency period through the active delay of metamorphosis^53^, may strongly contribute to fluctuations in levels of population connectivity at a finer spatial scale. However, it remains difficult to map directly how far and in what directions larvae disperse, thus complicating accurate stock definition.

Namely, both spatial analyses (Structure and DAPC) found relevant barriers to dispersal within the south Adriatic Sea area for both *Mullus* species, where south-eastern Adriatic populations were strongly differentiated genetically from the north-middle and south-western localities, suggesting high larval retention and confined dispersal across the geographic range of the South Adriatic Pit (from the Palagruža sill to Otranto) characterized by the narrow continental shelf and steep continental slope reaching a maximum depth of 1,270 m. Genetic differentiation of south-eastern populations were even more pronounced for red mullet. These findings agree with the hydrodynamic provinces in the Mediterranean from Lagrangian simulations and transport network reconstruction, where the eastern part of the Strait of Otranto was identified as a temporary stable gene flow barrier, even for larvae with long PLD (<30 days) and with an offshore larval distribution, such as *Mullus* species^58^. This also supported the north-south Adriatic subdivision already seen in some fish species^59^. In the present study, no genetic divergence was observed among populations from the western Adriatic coast and the northern Ionian Sea, in contrast to the outcomes of other works^18,25^ that revealed an isolation of Adriatic samples of red mullet from the neighbouring basins (Tyrrhenian Sea, Ionian Sea) and generally low structuring pattern within the Adriatic. The differences with our study could be due to the different resolution of molecular markers used or due to temporal fluctuation of population connectivity, highlighting the need to use temporal replicates when assessing population divergence patterns in marine fishes. Still, local oceanographic features support present gene flow findings, since the main sea current flows in the north–south direction along the western continental shelf^60^. It seems that the red mullet nurseries located in the south-western Adriatic Sea exploit the benefits of the continuous flux of the southern Adriatic water masses entering from the northern Ionian Sea^43^.

### Management implications

Valuable information on best techniques has been made available for delineating stocks^11,61^. Traditionally, assessment and management agents, such as the International Council for the Exploration of the Sea (ICES) and the GFCM, have delineated stock units by using geo-economic or political aspects related to the collection of fisheries data rather than based on population integrity^7^. Some flexibility in relation to new information can be seen nowadays. Namely, GFCM experts for Adriatic Sea pointed that management areas GSA17 and GSA 18 should not be maintained *a priori* as unique GFCM-GSA areas for joint stock assessment and management for *M. barbatus*, as conducted in previous years for *M. barbatus*^62^. They based their decision on the changing distribution of the red mullet population and the different selectivity of fleets exploiting this stock in both GSAs on the eastern and western Adriatic coast. Generally, *M. barbatus* seems to be in overexploitation but with relative high biomass although in some GSAs (as 18 and 25, sustainable exploitation of *M. babatus* is reported^45^ (see Table S4, Supplementary information).

Our results showed that a precautionary approach is needed when declaring subpopulations of certain species in small areas, such as the Adriatic Sea, to be one unique stock. Mixing data from sub-areas with marked differences in the state of exploitation of their stocks would increase the risk of population decline^63^. Surely, we are not averse to a re-evaluation of stock boundaries of GFCM-GSAs. Still, some authors suggested considering the entire set of current sub-areas rather than individual ones^8^, while others emphasized the need for using a holistic approach in definition of biological units (e.g. genetics, geography, marine habitats, fishing practices, socio-economy and policy implications)^61^, which is particularly important if a shift from mono-specific to an ecosystem approach is to be adopted in Mediterranean.

## Material and Methods

### Ethics statement

The methods involving animals in this study were conducted in accordance with the Laboratory Animal Management Principles of Croatia. All experimental protocols were approved by the Ethics Committee of the Institute of Oceanography and Fisheries.

### Sample collection, DNA extraction, and genotyping

A total of 1,347 adult individuals of *Mullus* spp. were collected from 14 different locations within the eastern, central and western Mediterranean regions, including the eastern Atlantic Sea (only in the case of *M. surmuletus*) (Fig. 1, Table S1, Supplementary information). To obtain fine population structure resolution in the Adriatic Sea, five populations of *M. surmuletus* and six populations of *M*. *barbatus* were sampled within the geographic range of the northern Adriatic to the Strait of Otranto. Samples were primarily obtained from fishing landings of local trawling gears at each location during 2015. Samples included a number of individuals ranging from 23 to 87, on the basis of location and species (Table 1). The distal portion of the pelvic fin of each collected fish was clipped and stored in 95% ethanol for subsequent genetic analysis.

Total genomic DNA from fins was extracted by proteinase K digestion, followed by standard phenol-chloroform extraction protocol. DNA quality and quantity were assessed by spectrophotometry (Eppendorf AG, Germany), and each sample was diluted to 10 ng μL^−1^ in DNAse/RNAse free water. Genetic analysis of all DNA samples was performed with a multiplex, comprising thirteen microsatellite markers of *Mullus* sp.: Mbar3, Mbar11, Mbar14, Mbar55, Mbar63, Mbar130, Mbar132 and Mbar133^64^ and Mbarb002, Mbarb051, Mbarb054, Mbarb064 and Mbarb101^65^. The primer dies were carefully set up avoiding similar allele size overlapping. Using the Multiplex PCR kit (Qiagen, Germany), amplification of the loci was run in 12.5 μL reactions, containing 10 ng DNA, on an Eppendorf Mastercycler Nexus Gx2 thermal cycler. Final concentrations of all primers were uniformly set at 0.2 µM. Thermal cycling consisted of an initial denaturation step at 95 °C for 5 min followed by 26 cycles of: denaturation at 95 °C, annealing at 57 °C, and extension at 72 °C for 30 s, 1.5 min, and 30 s, respectively. A final cycle at 60 °C for 30 min was performed. Fragments were separated on an ABI3130 automated sequencer (Applied Biosystems) while peak height values for each microsatellite allele were scored manually by two different persons using GeneMapper v.3.5 software (Applied Biosystems).

### Hardy-Weinberg Equilibrium, Linkage Disequilibrium and Null Alleles

Scored alleles were checked for genotyping errors using MICROCHECKER 2.2.3^66^ while the presence and frequency of null alleles were additionally examined by FreeNA^19^ following the Expectation Maximization (EM) algorithm. FreeNA was used to compute the FST statistic, both with exclusion and inclusion of the ENA (Excluding Null Alleles) correction method, considering that the presence of null alleles can result in an overestimation of population genetic differentiation. The bootstrap 95% confidence intervals (CI) for the global FST values were calculated using 50,000 replicates over loci.

GENEPOP v.3.1b^67^ was used to perform an exact test for deviations from Hardy-Weinberg equilibrium by testing heterozygote deficiency and heterozygote excess in the microsatellite data. The linkage disequilibrium (LD) test for all pairs of loci was also performed in GENEPOP v.3.1b. Exact P-values for the individual population or locus tests were estimated using the Markov Chain algorithm (10,000 dememorization steps, 100 batches and 5000 iterations) and the significance of HWE and LD values were adjusted by Bonferroni correction.

### Genetic diversity, test of demographic changes and effective population size

Observed (*H*o) and expected (*H*e) heterozygosity was calculated in GENEPOP v.3.1b. The mean number of alleles per locus (*A*) and mean effective number of alleles across loci (*A*e) were calculated using POPGENE v.1.32^68^, while allelic richness *(A*r) and inbreeding coefficient (*F*_*IS*_) were calculated using FSTAT v.2.9.3^69^.

Genetic evidence for a recent reduction in local population size were tested by Heterozygosity excess^70^ and M ratio^39^ methods. Heterozygosity excess tests were performed with the program BOTTLENECK 1.2.02^71^ by the Infinite Allele model (IAM) and Two-Phased mutation model (TPM), incorporating 90% of single-step mutations and 10% of variance among multiple steps. Statistical significance was evaluated by Wilcoxon signed-rank test from 10,000 simulation replicates. Secondly, the software packages MPVal and CriticalM^39^ were used to trace for signatures of a bottleneck by the M-ratio method. The total number of alleles (*k*) divided by overall range in allele size (*r*) produces the ratio (M), which is expected to be smaller in recently reduced populations than in populations under mutation-drift equilibrium^39^. The simulated analysis using a constant mutation rate at μ of 5 × 10^−4^ was run assuming a TPM model with parameters *p*_*s*_ (proportion of one-step mutations) = 0.88 and *Δ*_*g*_ (average size of multistep mutations) = 2.8^39^. The empirical M values were compared to 95% critical values (*M*_c_) below which a bottleneck is evident, derived from 10,000 simulations of stable populations using the program Critical_m. Ancestral theta (Θ) was set at three different values (0.5, 1, 10) corresponding to a pre-bottleneck effective population size of 250, 500 and 5000, respectively.

The program NeEstimator 2 was used to estimate contemporary effective population size (*N*_e_) using the single-sample linkage disequilibrium method^72^ for each of the 25 populations examined. Populations with a small sample size below 30 individuals (i.e. IS_LS and CP_LS) were not analysed. Low frequency alleles ≤0.02 were excluded from the analysis to minimize potential bias caused by rare alleles.

### Genetic differentiation and population structuring

POWSIM^73^ was used to assess the statistical power of tests for genetic homogeneity on the applied set of markers and sample sizes. The interpopulation level of gene flow was quantified by estimating pair-wise and global *F*_ST_ values as a weighted average over 11 loci in ARLEQUIN v.3.5, where statistical significance was assessed following 10,000 permutations.

Analysis of the genetic structure of sampled populations was performed using the Bayesian-clustering program STRUCTURE 2.3^74^. In addition, the Discriminant Analysis of Principal Components (DAPC) was employed as a non model-based method recently developed and implemented in the Adegenet package^75^ for R software^76^. STRUCTURE analysis was conducted on the full sample dataset using the admixture ancestry model, correlated allele frequencies, a burn-in period of 50^3^ iterations followed by 50^4^ MCMC steps and *K* values from 1 to the maximal number of sampled groups with 20 replicates each. To assess the most likely number of clusters, ln *P*(*D*) and ∆*K* values were estimated in Structure Harvester 0.6.93^77^. CLUMPP v.1.1.2^78^ was used to assess the average pairwise similarity of runs and plots of the clusters produced in Microsoft Excel. Finally, to obtain an easily interpretable representation of the estimated admixture proportions in geographic space for the Adriatic Sea, the membership coefficient matrix (*individual Q-matrix*) was interpolated using the maps function from POPSutilities.R^79^ in R 3.3.3 software and by using the *max* option where only the cluster with the maximal local contribution to ancestry is represented at each geographic point of the map.

To explore how the genetic variation was partitioned among sampling localities, Discriminant Analysis of Principal Components was executed by the function dapc using the sampling locality as a prior^75^. The number of Principal Components (PCs) retained was optimized using the function xvalDapc.

To test the correlation between genetic and geographic distances, isolation by distance (IBD) analysis was performed with the package *Adegenet* in R. Mantel’s test was used to check for correlation between Edwards’ distances and Euclidean geographic distances among populations with the *mantel.randtest* function (1000 permutations). To test whether the correlation between genetic and geographic distances is a result of a continuous or distant patchy cline of genetic differentiation, local densities of distances to disentangle both processes were plotted using a 2-dimensional kernel density estimation (function kde2d). The results were displayed with a customized colour palette using image in the MASS package^80,81^.

### Data availability

The full dataset of genotypes has been deposited into GenoBase of Institute of Oceanography and Fisheries (http://jadran.izor.hr/~tsegvic/aquapop/GenoBase.html) and is available from the corresponding authors on reasonable request.

## Electronic supplementary material

Supplementary Information
