# Supplementary material for: Evidence of subtle genetic structure in the sympatric species Mullus barbatus and Mullus surmuletus (Linnaeus, 1758) in the Mediterranean Sea
Source: Sci Rep. 2018 Jan 12;8:676. doi: 10.1038/s41598-017-18503-7 (PMC5766513; doi:10.1038/s41598-017-18503-7)
Supplement: Supplementary file 1 — Supplementary Information [file 41598_2017_18503_MOESM1_ESM.pdf]

Evidence of subtle genetic structure in the sympatric species *Mullus barbatus* and *Mullus surmuletus* (Linnaeus, 1758) in the Mediterranean Sea

Sanja Matić-Skoko[1], Tanja Šegvić-Bubić[1]*, Ivana Mandić[1], David Izquierdo-Gomez[2], Enrico Arneri[3], Pierluigi Carbonara[4], Fabio Grati[5], Zdravko Ikica[6], Jerina Kolitari[7], Nicoletta Milone[3], Paolo Sartor[8], Giuseppe Scarcella[5], Adnan Tokaç[9] and Evangelos Tzanatos[10]

[1] *Institute of Oceanography and Fisheries, Šetalište I. Meštrovića 63, 21000, Split, Croatia*
[2] *Production Biology Department, Nofima AS, P.O. Box 6122, 9291 Tromsø, Norway*
[3] *FAO AdriaMed Project, Viale delle Terme di Caracalla, 00153 Roma, Italy*
[4] *COISPA Tecnologia & Ricerca - Stazione Sperimentale per lo Studio delle Risorse del Mare Via dei Trulli 18/20 70126 Bari, Italy*
[5] *ISMAR-CNR, Institute of Marine Sciences of the Italian National Research Council, Largo Fiera della Pesca 2, 60125, Ancona, Italy*
[6] *Institute of Marine Biology, University of Montenegro, P. Fah 69, 85330, Kotor, Montenegro*
[7] *Aquaculture and Fishery Laboratory, Agricultural University of Tirana*
[8] *CIBM Centro Interuniversitario Biologia Marina ed Ecologia Applicata "G. Bacci", Viale Nazario Sauro 4, 57128 Livorno, Italy*
[9] *Ege University, Faculty of Fisheries, 35100 Bornova, Izmir, Turkey*
[10] *Department of Biology, University of Patras, 26504, Patras, Greece*

**Corresponding author: tel: +385 21 408 000, fax: +385 21 358 650; e-mail: tsegvic@izor.hr*

Supplement Table S1. Origin of the studied samples of *Mullus barbatus and Mullus surmuletus*, and corresponding number of specimens amplified with the microsatellites markers and included in the analyses.

Species	Sea	Region, Country (GSA)	Geographic coordinates		Code	N
M. barbatus<br>*M. surmuletus*	Mediterranean	North Adriatic, Croatia (GSA17)	44.943815°	13.605487°	CRO_NAS	48<br>50
M. barbatus<br>*M. surmuletus*	Mediterranean	Middle Adriatic, Croatia (GSA17)	43.568418°	15.769985°	CRO_MAS	66<br>54
M. barbatus<br>*M. surmuletus*	Mediterranean	South Adriatic, Croatia (GSA17)	42.615188°	17.779310°	CRO_SAS	50<br>49
M. barbatus	Mediterranean	North Adriatic, Italy (GSA17)	45.009044°	13.004869°	ITA_NAS	49
M. barbatus,<br>*M. surmuletus*	Mediterranean	Middle Adriatic, Italy (GSA17)	43.750132°	13.757831°	ITA_MAS	50<br>47
M. barbatus,<br>*M. surmuletus*	Mediterranean	South Adriatic, Italy (GSA17)	41.504365°	16.810572°	ITA_SAS	47<br>42
M. barbatus,<br>*M. surmuletus*	Mediterranean	Tyrrhenian Sea, Italy (GSA9)	41.503912°	11.065175°	ITA_TS	49<br>45
M. barbatus,<br>*M. surmuletus*	Mediterranean	South Adriatic, Montenegro (GSA18)	42.266861°	18.618152°	MN_AS	87<br>31
M. barbatus	Mediterranean	South Adriatic, Albania (GSA18)	41.162552°	19.259209°	AL_AS	67
M. barbatus,<br>*M. surmuletus*	Mediterranean	Ionian Sea, Greece (GSA20)	38.171978°	22.651481°	GR_IS	59<br>59
M. barbatus,<br>*M. surmuletus*	Mediterranean	Aegean Sea, Turkey (GSA22)	37.947463°	26.720771°	TR_AS	48<br>49
M. barbatus,<br>*M. surmuletus*	Mediterranean	Levantine Sea, Israel (GSA27)	32.335961°	34.363010°	IS_LS	23<br>15
M. barbatus,<br>*M. surmuletus*	Mediterranean	Levantine Sea, Cyprus (GSA25)	34.429656°	32.220743°	CP_LS	31<br>49
M. barbatus,<br>*M. surmuletus*	Mediterranean	Balearic Sea, Spain (GSA6)	38.572230°	0.637080°	SP_BS	46<br>41
M. surmuletus	N-E Atlantic	Portugal	40.154904°	9.21806°	PT_AO	68

Supplement Table S2. Summery statistics of 13 microsatellite loci among samples of *Mullus barbatus* and *Mullus surmuletus*.

M.barbatus		**Sampling code**													
**Locus**		**CRO NAS**	**CRO MAS**	**CRO SAS**	**ITA NAS**	**ITA MAS**	**ITA SAS**	**ITA TS**	**MN AS**	**AL AS**	**GR IS**	**TR LS**	**IS LS**	**CP LS**	**SP BS**
	n	48	66	50	49	50	47	49	87	67	59	48	23	29	46
Mbar	nA	13	11	10	10	10	8	10	11	9	11	8	8	9	10
132	HO	0.771	0.848	0.720	0706	0.660	0.787	0.715	0.655	0.672	0.763	0.750	0.739	0.862	0.543
	HE	0.734	0.769	0.710	0.717	0.632	0.734	0.733	0.652	0.711	0.732	0.746	0.734	0.769	0.636
	n	47	66	50	49	50	45	48	87	65	59	48	22	20	46
Mbar	nA	31	33	31	27	30	32	31	36	35	31	31	20	20	28
14	HO	0.914	0.924	0.860	0.857	0.820	0.867	0.833	0.851	0.923	0.864	0.896	0.772	0.850	0.804
	HE	0.963	0.939	0.948	0.949	0.960	0.968	0.955	0.957	0.957	0.952	0.949	0.948	0.958	0.938
	n	48	66	50	49	50	47	49	87	67	59	48	23	31	46
Mbar	nA	6	6	6	4	6	6	6	6	6	6	6	6	7	5
064	HO	0.562	0.500	0.500	0.571	0.540	0.532	0.530	0.506	0.657	0.542	0.688	0.565	0.548	0521
	HE	0.521	0.511	0.502	0.531	0.544	0.589	0.576	0.558	0.559	0.558	0.614	0.581	0.580	0.490
	n	48	66	50	49	50	47	49	87	67	59	48	23	31	46
Mbar	nA	10	8	8	9	7	9	7	9	11	10	9	9	7	10
051	HO	0.625	0.606	0.640	0.673	0.620	0.787	0.694	0.724	0.657	0.661	0.625	0.609	0.580	0.696
	HE	0.656	0.602	0.611	0.703	0.599	0.787	0.665	0.688	0.666	0.631	0.675	0.726	0.652	0.688
	n	48	66	50	49	50	47	49	87	67	59	48	23	31	46
Mbar	nA	8	9	8	10	9	9	7	9	9	10	8	7	9	11
002	HO	0.708	0.666	0.700	0.877	0.660	0.829	0.633	0.701	0.701	0.627	0.729	0.696	0.742	0.696
	HE	0.724	0.730	0.690	0.771	0.714	0.725	0.702	0.708	0.712	0.761	0.673	0.723	0.816	0.739
	n	48	64	50	49	49	45	47	85	66	59	47	20	21	45
Mbar	nA	16	15	14	15	12	12	12	14	14	10	16	10	9	14
63*	HO	0.687	0.609	0.690	0.796	0.571	0.800	0.638	0.718	0.591	0.610	0.681	0.650	0.571	0.666
	HE	0.852	0.851	0.857	0.859	0.830	0.858	0.853	0.843	0.842	0.841	0.889	0.877	0.872	0.891
	n	48	65	50	49	50	47	49	87	67	59	48	23	31	46
Mbar	nA	2	2	2	2	2	2	2	3	3	2	2	3	3	1
101*	HO	0.000	0.000	0.000	0.000	0.000	0.000	0.000	0.012	0.015	0.017	0.000	0.000	0.032	0.783
	HE	0.491	0.143	0.424	0.247	0.298	0.384	0.679	0.034	0.044	0.017	0.041	0.166	0.206	0.878
	n	48	65	50	49	50	45	47	87	67	58	48	23	27	46
Mbar	nA	16	17	17	17	17	17	18	19	19	14	13	13	14	13
55	HO	0.708	0.800	0.850	0.755	0.840	0.867	0.851	0.839	0.851	0.741	0.792	0.696	0.778	0.782
	HE	0.889	0.876	0.889	0.877	0.911	0.916	0.921	0.888	0.900	0.904	0.879	0.922	0.899	0.877
	n	40	55	44	41	43	37	46	73	61	50	44	18	21	36
Mbar	nA	11	13	11	13	12	17	10	11	8	11	12	7	9	10
130*	HO	0.250	0.345	0.254	0.293	0.302	0.479	0.174	0.164	0.229	0.100	0.227	0.056	0.142	0.305
	HE	0.836	0.829	0.782	0.788	0.796	0.868	0.737	0.699	0.789	0.819	0.837	0.678	0.829	0.825
Mbar	n	48	66	50	49	50	46	49	87	66	58	48	22	23	46
56	nA	9	8	8	7	8	8	8	8	11	9	9	6	6	7
	HO	0.645	0.636	0.740	0.735	0.660	0.674	0.673	0.829	0.621	0.689	0.688	0.773	0.435	0.609
	HE	0.692	0.663	0.745	0.712	0.664	0.671	0.670	0.696	0.700	0.718	0.737	0.683	0.669	0.682
	n	32	41	33	35	36	30	30	74	56	43	47	20	20	35
Mbar	nA	19	20	16	18	18	17	19	28	23	14	21	14	13	21
3*	HO	0.281	0.341	0.424	0.171	0.222	0.233	0.200	0.338	0.500	0.256	0.298	0.250	0.250	0.257
	HE	0.946	0.933	0.919	0.927	0.926	0.902	0.939	0.942	0.944	0.894	0.928	0.919	0.892	0.942
	n	48	65	50	49	50	46	49	87	67	59	48	22	26	46
Mbar	nA	13	13	13	12	16	12	9	13	12	12	11	9	11	12
11	HO	0.666	0.575	0.600	0.653	0.640	0.652	0.551	0.609	0.955	0.695	0.583	0.682	0.692	0.608
	HE	0.670	0.652	0.668	0.626	0.688	0.716	0.618	0.622	0.553	0.702	0.660	0.682	0.674	0.650
	n	47	65	50	49	50	45	49	84	64	59	48	22	19	45
Mbar	nA	18	18	19	14	15	14	19	18	16	17	19	12	7	18
133	HO	0.766	0.677	0.740	0.639	0.720	0.622	0.735	0.667	0.625	0.729	0.813	0.591	0.579	0.777
	HE	0.890	0.825	0.852	0.882	0.851	0.806	0.837	0.859	0.829	0.856	0.859	0.845	0.665	0.842

M. surmuletus		Sampling code												
**Locus**	**Indices**	**CRO NAS**	**CRO MAS**	**CRO SAS**	**ITA MAS**	**ITA SAS**	**ITA TS**	**MN AS**	**GR IS**	**TR LS**	**IS LS**	**CP LS**	**SP BS**	**PT AO**
	n	50	54	49	47	42	45	31	59	49	15	49	41	68
Mbar	nA	14	12	14	11	12	13	13	13	15	7	14	13	16
132	HO	0.800	0.722	0.816	0.872	0.928	0.822	0.806	0.673	0.816	0.800	0.796	0.805	0.779
	HE	0.841	0.818	0.973	0.833	0.835	0.856	0.852	0.825	0.814	0.752	0.875	0.853	0.855
	n	50	54	48	47	42	45	31	59	49	15	49	41	68
Mbar	nA	22	23	22	20	21	25	22	22	20	10	22	21	26
14	HO	0.880	0.888	0.937	0.936	0.871	0.977	1.000	0.763	0.939	0.933	0.877	0.951	0.897
	HE	0.939	0.919	0.941	0.936	0.917	0.944	0.950	0.917	0.934	0.816	0.911	0.932	0.926
	n	50	54	49	47	42	45	31	59	49	15	49	41	68
Mbar	nA	5	4	3	2	2	4	5	6	2	2	5	2	4
064	HO	0.200	0.148	0.265	0.128	0.357	0.244	0.226	0.288	0.184	0.133	0.347	0.097	0.162
	HE	0.302	0.173	0.282	0.121	0.297	0.223	0.343	0.312	0.201	0.129	0.371	0.178	0.255
	n	50	53	49	47	42	45	31	59	47	15	49	41	68
Mbar	nA	16	17	18	16	14	16	12	17	17	9	13	17	16
051	HO	0.660	0.830	0.755	0.745	0.715	0.777	0.645	0.831	0.875	0.667	0.633	0.732	0.618
	HE	0.899	0.909	0.894	0.895	0.870	0.909	0.899	0.883	0.912	0.696	0.876	0.906	0.876
	n	50	54	49	47	42	45	31	59	49	15	49	41	68
Mbar	nA	10	9	9	8	7	9	8	9	7	6	10	8	9
002	HO	0.660	0.648	0.775	0.766	0.786	0.777	0.742	0.746	0.714	0.600	0.775	0.658	0.750
	HE	0.712	0.772	0.787	0.772	0.691	0.757	0.827	0.767	0.779	0.825	0.750	0.776	0.756
	n	50	54	49	47	42	45	31	59	49	15	49	41	68
Mbar	nA	16	18	19	15	18	16	12	18	17	9	15	15	17
63	HO	0.700	0.833	0.837	0.808	0.833	0.689	0.677	0.798	0.796	0.533	0.551	0.805	0.823
	HE	0.893	0.873	0.889	0.886	0.888	0.868	0.877	0.879	0.879	0.887	0.874	0.874	0.861
	n	48	51	49	47	42	45	30	59	49	15	49	41	67
Mbar	nA	7	7	6	6	6	8	7	8	6	4	6	9	7
101	HO	0579	0.726	0.653	0.617	0.786	0.644	0.567	0.695	0.592	0.467	0.715	0.683	0.522
	HE	0.684	0.645	0.704	0.686	0.656	0.692	0.708	0.680	0.661	0.494	0.627	0.714	0.572
	n	50	54	49	46	42	45	31	59	49	15	49	41	68
Mbar	nA	12	7	8	9	9	7	12	10	10	3	8	6	9
55	HO	0780	0.685	0.633	0.739	0.857	0.644	0.742	0.644	0.755	0.667	0.775	0.658	0.521
	HE	0.759	0.688	0.716	0.750	0.718	0.730	0.749	0.708	0.746	0.536	0.738	0.717	0.703
	n	45	37	41	37	42	37	30	52	45	15	40	36	51
Mbar	nA	16	9	12	13	10	9	10	14	10	7	15	13	17
130*	HO	0.244	0.063	0.171	0.270	0.214	0.270	0.300	0.288	0.133	0.267	0.125	0.139	0.216
	HE	0.873	0.744	0.825	0.783	0.766	0.624	0.814	0.821	0.692	0.827	0.863	0.809	0.856
	n	47	54	49	47	42	45	31	59	49	14	47	41	68
Mbar	nA	7	7	7	7	7	7	8	7	7	6	7	6	7
56	HO	0.851	0.833	0.673	0.787	0.833	0.889	0.839	0.831	0.837	0.643	0.766	0.756	0.809
	HE	0.806	0.814	0.777	0.761	0.763	0.798	0.800	0.779	0.806	0.754	0.817	0.757	0.766
	n	33	48	46	47	42	44	30	58	48	10	37	33	64
Mbar	nA	8	9	7	8	7	8	9	8	5	3	7	6	5
3	HO	0.333	0.500	0.500	0.638	0.595	0.341	0.367	0.431	0.431	0.500	0.297	0.424	0.312
	HE	0.415	0.523	0.508	0.550	0.545	0.372	0.481	0.423	0.402	0.426	0.371	0.394	0.302
	n	42	50	47	47	42	45	29	58	48	10	19	31	55
Mbar	nA	9	7	9	8	7	8	12	11	9	7	14	7	7
11	HO	0.381	0.600	0.808	0.638	0.762	0.711	0.586	0.741	0.687	0.700	0.526	0.581	0.545
	HE	0.759	0.730	0.785	0.777	0.684	0.744	0.821	0.768	0.783	0.863	0.824	0.743	0.699
	n	45	49	49	45	41	43	29	57	48	14	40	39	64
Mbar	nA	11	14	13	10	13	13	13	16	12	7	12	12	12
133*	HO	0.111	0.224	0.143	0.311	0.439	0.302	0.448	0.281	0.229	0.428	0.325	0.231	0.172
	HE	0.866	0.907	0.874	0.874	0.884	0.907	0.892	0.900	0.884	0.873	0.877	0.868	0.882

Supplement Table S3. Bottleneck results of one-tail Wilcoxon test for heterozygote excess and observed values of the *M*-ratio averaged over the number of polymorphic microsatellite loci for each *Mullus barbatus* (a) and *Mullus surmuletus* (b) contemporary sample considered in this study. For different ancestral theta (0.5, 1, 10), the *p*-value at which a bottleneck occurred is presented. NS = not significant.

a) *Mullus barbatus*	Bottleneck		M	M ratio (*P*-value)		
	IAM	TPM(90)		Θ = 0.5	Θ = 1	Θ = 10
CRO_NAS	Ns	ns	0.754	ns	ns	ns
CRO_MAS	Ns	ns	0.763	ns	ns	ns
CRO_SAS	Ns	ns	0.796	ns	ns	ns
ITA_NAS	Ns	ns	0.792	ns	ns	ns
ITA_MAS	Ns	ns	0.73	< 0.05	ns	ns
ITA_SAS	Ns	ns	0.78	ns	ns	ns
ITA_TS	Ns	ns	0.806	ns	ns	ns
MN_AS	Ns	ns	0.806	ns	ns	ns
AL_AS	Ns	ns	0.77	ns	ns	ns
GR_IS	< 0.05	ns	0.741	< 0.001	< 0.001	< 0.001
TR_AS	< 0.05	ns	0.729	< 0.001	< 0.001	< 0.001
SP_BS	Ns	ns	0.773	ns	ns	ns

b) *Mullus surmuletus*	Bottleneck		M	M ratio (*P*-value)		
	IAM	TPM(90)		Θ = 0.5	Θ = 1	Θ = 10
CRO_NAS	Ns	ns	0.723	< 0.03	ns	ns
CRO_MAS	Ns	ns	0.768	ns	ns	ns
CRO_SAS	Ns	ns	0.76	ns	ns	ns
ITA_MAS	Ns	ns	0.806	ns	ns	ns
ITA_SAS	Ns	ns	0.8	ns	ns	ns
ITA_TS	Ns	ns	0.765	ns	ns	ns
MN_AS	Ns	ns	0.695	< 0.02	ns	ns
GR_IS	Ns	ns	0.732	ns	ns	ns
TR_AS	< 0.01	ns	0.688	< 0.001	< 0.01	ns
CP_LS	Ns	ns	0.764	ns	ns	ns
SP_BS	Ns	ns	0.76	ns	ns	ns
PT_AO	Ns	ns	0.703	< 0.02	ns	ns

Supplement Table S4. GFCM stock assessment outcomes for *Mullus barbatus* in 2016[45] in relation with observed heterozygosity and M ratio values

GSA	*Mullus barbatus*			M-ratio
	F_{cur}	State	Observed Heterozygosity	
GSA 6 (Northern Spain)	$F_{cur} > F_{0.1}$	Overexploitation	0.67±0.1	0.773
GSA 9 (Ligurian Sea and Northern Tyrrhenian Sea)	$F_{cur} > F_{0.1}$	High overfishing	0.68±0.1	0.806
GSA 17 (Northern Adriatic Sea)	$F_{cur} > F_{0.1}$	High overfishing	0.68±0.1<br>0.70±0.9(CRO)<br>0.67±0.1<br>0.73±0.1 (ITA)	0.754-0.763 (CRO)<br>0.73-0.792 (ITA)
GSA 18 (Southern Adriatic Sea)	$F_{cur} < F_{0.1}$	Sustainable exploitation	0.70±0.1(CRO)<br>0.74±0.1 (ITA)	0.796 (CRO)<br>0.78 (ITA)
GSA 20 (Eastern Ionian Sea)		n.a.	0.69±0.1	0.741
GSA 22 (Aegean Sea)		n.a.	0.72±0.1	0.729
GSA 25 (Cyprus)	$F_{cur} < F_{0.1}$	Sustainable exploitation	0.66±0.1	-
GSA 27 (Eastern Levant Sea)		n.a.	0.67±0.1	-

F_{cur} – current fishing mortality; $F_{0.1}$ – the fishing mortality rate at which the slope of the yield-per-recruit curve is only one-tenth the slope of the curve at its origin

Supplement Figures

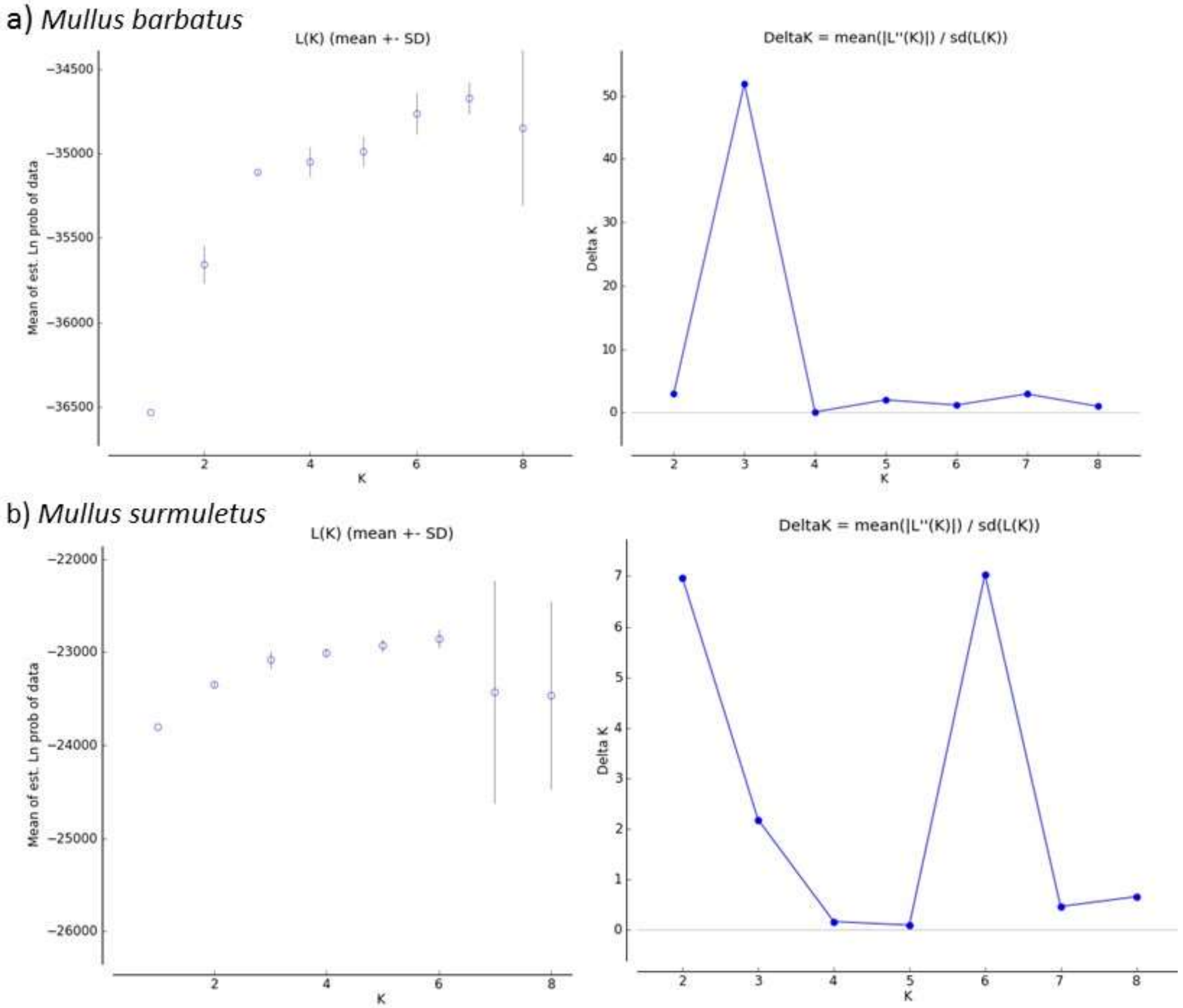

Suppl. Figure S1. Inference of the number of clusters in *Mullus barbatus* (a) and *Mullus surmuletus* (b) populations data. The first plot represents the values for the mean likelihood of each genetic cluster (K), where the error bar represents one standard deviation, while the second plot represents values of Delta K calculated as the mean of the second-order rate of change in the likelihood of K divided by the standard deviation of the likelihood of K.

a) *Mullus barbatus*

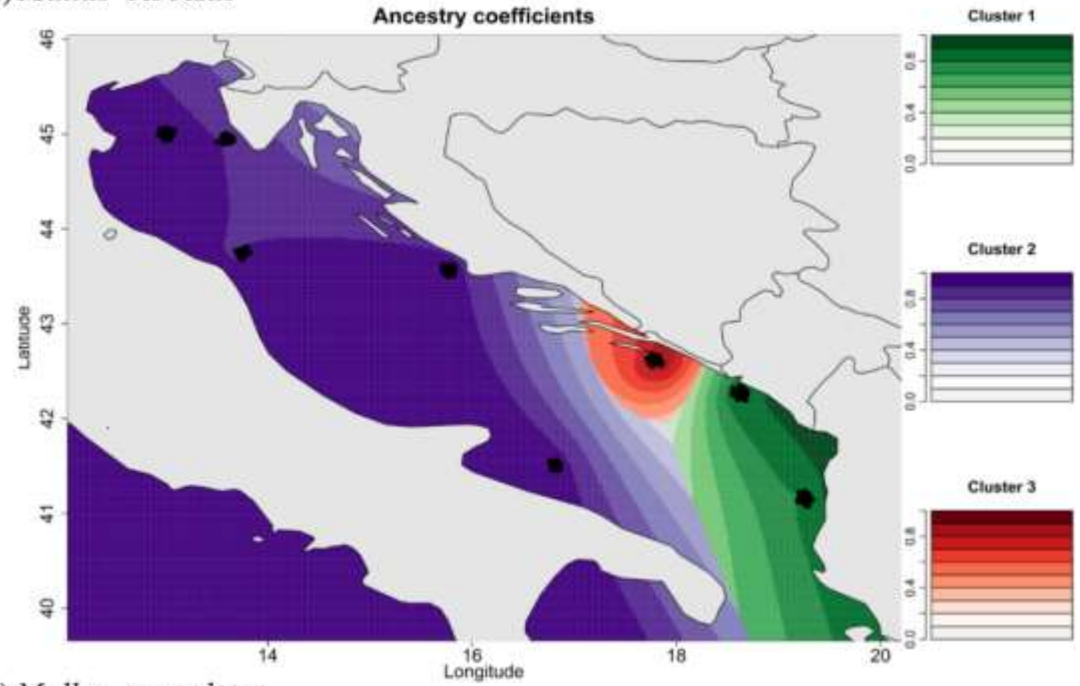

b) *Mullus surmuletus*

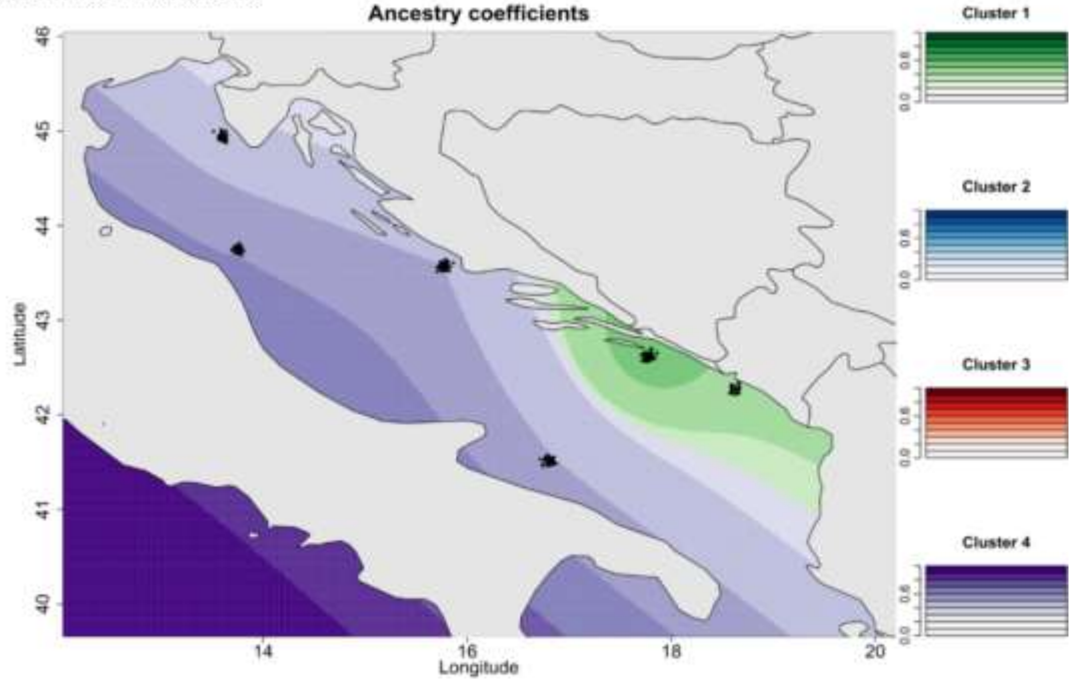

Suppl. Figure S2. STRUCTURE *Q* scores of *Mullus barbatus* (a) and *Mullus surmuletus* (b) individuals from Adriatic Sea displayed spatially by universal kriging. *Q* scores were represented on the ETOPO1 map (produced by NOAA and freely available as indicated here: https://www.ngdc.noaa.gov/mgg/global/dem_faq.html#sec-2.4[82]) using the maps function from the POPSutilities.R[79] in R 3.3.3 software and by using the *max* option where only the cluster with the maximal local contribution to ancestry is represented at each geographic point of the map.
